# Effects of drip irrigation on yield, soil fertility and soil enzyme activity of different potato varieties in Northwest China

**DOI:** 10.3389/fpls.2023.1240196

**Published:** 2023-08-29

**Authors:** Fan Zhang, Mengru Chen, Jintao Fu, Xiangzhu Zhang, Yuan Li, Yingying Xing

**Affiliations:** College of Life Sciences, Yan’an University, Yan’an, Shaanxi, China

**Keywords:** potato, soil fertility, soil enzyme activity, yield, variety

## Abstract

The effects of different irrigation and fertilization on potato yield, soil fertility and soil enzyme activity of different varieties under drip irrigation fertilization mode were studied, which could provide support for selecting the best varieties in Northwest China. Three factors and three levels orthogonal experimental design method, a total of nine treatments. The three irrigation levels were W1 (100% crop evapotranspiration (ET_C_)), W2 (80% ET_C_) and W3 (60% ET_C_). The three fertilization levels were F1 (N-P_2_O_5_-K_2_O, 240-120-300 kg ha^−1^), F2 (180-90-225 kg ha^−1^) and F3 (120-60-150 kg ha^−1^). The three varieties were V1 (Feiuritar), V2 (Longshu7) and V3 (Qingshu 9). The results showed that different irrigation and fertilization had significant effects on potato yield, soil fertility and soil enzyme activity in root zone. The highest yield of T5 (80%ET_C_, 180-90-225 kg ha^−1^, Qingshu 9) was 49,222.3 kg ha^−1^. With the increase of fertilizer application rate, potato yield and soil enzyme activity in root zone increased first and then decreased, but soil electrical conductivity (SEC), soil nitrate-N content (SNNC), soil alkali-hydrolyzable nitrogen content (SAHC), soil available potassium (AK), soil available phosphorus (AP), soil ammonium-N content (SANC) and soil organic matter (SOM) in root zone increased continuously. The yield, soil catalase activity, soil urease activity and soil sucrase activity at W2 were 2.81% and 22.2%, 1.84% and 7.04%, 8.26% and 9.62%, 5.34% and 13.36% higher than those at W1 and W3, respectively. The overall trend of soil water content, soil nutrient content and enzyme activity in root zone was 0–20 cm >20–40 cm >40–60 cm soil layer. There were many soil factors affecting tuber yield, among which soil enzyme activity, pH value and root zone conductivity were the key factors. The results showed that T5 (80%ET_C_, 180-90-225 kg ha^−1^, Qingshu 9) was the best treatment to improve soil enzyme activity and yield.

## 1 Introduction

Potato (*Solanum tuberosum* L.) is the world’s fourth largest food crop after maize, wheat and rice, and plays an important role in ensuring food security (Riao et al., 2022). Due to the unique climatic factors and soil conditions, northern Shaanxi plays an irreplaceable role in potato production (Xing et al., 2020). However, there are many problems in potato production in this region, such as over irrigation and fertilization, improper selection of varieties (Wang et al., 2019b; Wang et al., 2021b; Zhou et al., 2022). These problems have worsened the soil micro-ecosystem and limited the development of the potato industry in the region. It is very important to choose reasonable planting varieties and irrigation and fertilization system for the healthy, green and sustainable development of potato industry in this region. Soil enzyme activity is an important index affecting soil nutrient cycling, which can objectively evaluate soil nutrient status (Wang et al., 2019c; Xiao et al., 2023). It is important to study the dynamic change of soil enzyme activity in root zone for crop yield increase and soil quality evaluation (Paul et al., 2016; Gao et al., 2022). The responses of different irrigation rates, fertilization rates and varieties to soil enzyme activities and nutrients in crop root areas were also different (Rajanna et al., 2022; Xing et al., 2022; Ai et al., 2023). However, the activities of soil urease activity, soil catalase activity and soil sucrase activity in the root zone of soil were significantly decreased when appropriate fertilizer was applied (Xing et al., 2022). Drip irrigation under mulch can significantly improve soil enzyme activity, rhizosphere micro-environment, crop yield and quality (Kang et al., 2021; Kaur et al., 2023). The yield and root layer soil factor analysis of local potato varieties showed that there were significant differences in yield, physical and chemical indexes and enzyme activities of different potato varieties and soil nutrients were closely related to root layer enzyme activities (Wang et al., 2019a; Cheng et al., 2021).

At present, most studies focus on the effects of single or double factors such as irrigation amount, fertilizer rate and variety on crop yield, soil nutrients, and soil enzyme activities. There are some differences in the results of different studies. In agricultural production practice, irrigation, fertilizer and varieties need to be managed synchronously. However, studies on the effects of the three factors on potato yield, soil nutrients and enzyme activities are scarce, especially in arid regions of northwest China. Therefore, this experiment adopted the orthogonal experimental design method to explore the effects of drip irrigation fertilization mode on dynamic changes of potato yield, soil fertility and soil enzyme activity of different varieties in arid regions of northwest China. At the same time, correlation analysis and partial least square method were used to analyze the coupling relationship between soil nutrients and enzyme activities in the root zone, and screen the important soil factors affecting potato yield in this area. In order to provide scientific basis for selecting the best varieties of efficient water and fertilizer management mode in this area.

## 2 Materials and methods

### 2.1 Site description

Field trials was performed in Yan’an city, Shaanxi Province, China (N36°39′, E109°11′), at an altitude of 1,100 m. The experiments were performed at Yan’an University Experimental Station during the potato growing season in 2022. The region belongs to temperate arid and semi-arid continental monsoon climate. The annual average air temperature is 8.9°C, annual average precipitation is 473 mm, average annual evaporation is 1800 mm and the no-frost cycle ranges from 150 to 200 days. The soil at the test site was sandy loam. Before the implementation of the test, the soil bulk density of cultivated soil (0–60 cm) was 1.28 g cm^−3^, field water capacity was 7.89%, soil pH value was 8.43 (1:5 soil: water), soil ammonium nitrogen content was 3.34 mg kg^−1^, soil nitrate nitrogen content was 13.30 mg kg^−1^, soil available phosphorus content was 21.43 mg kg^−1^, soil organic matter content was 7.66 g kg^−1^ and soil available potassium content was 98.3 mg kg^−1^.

### 2.2 Experimental design

**Figure 1:**
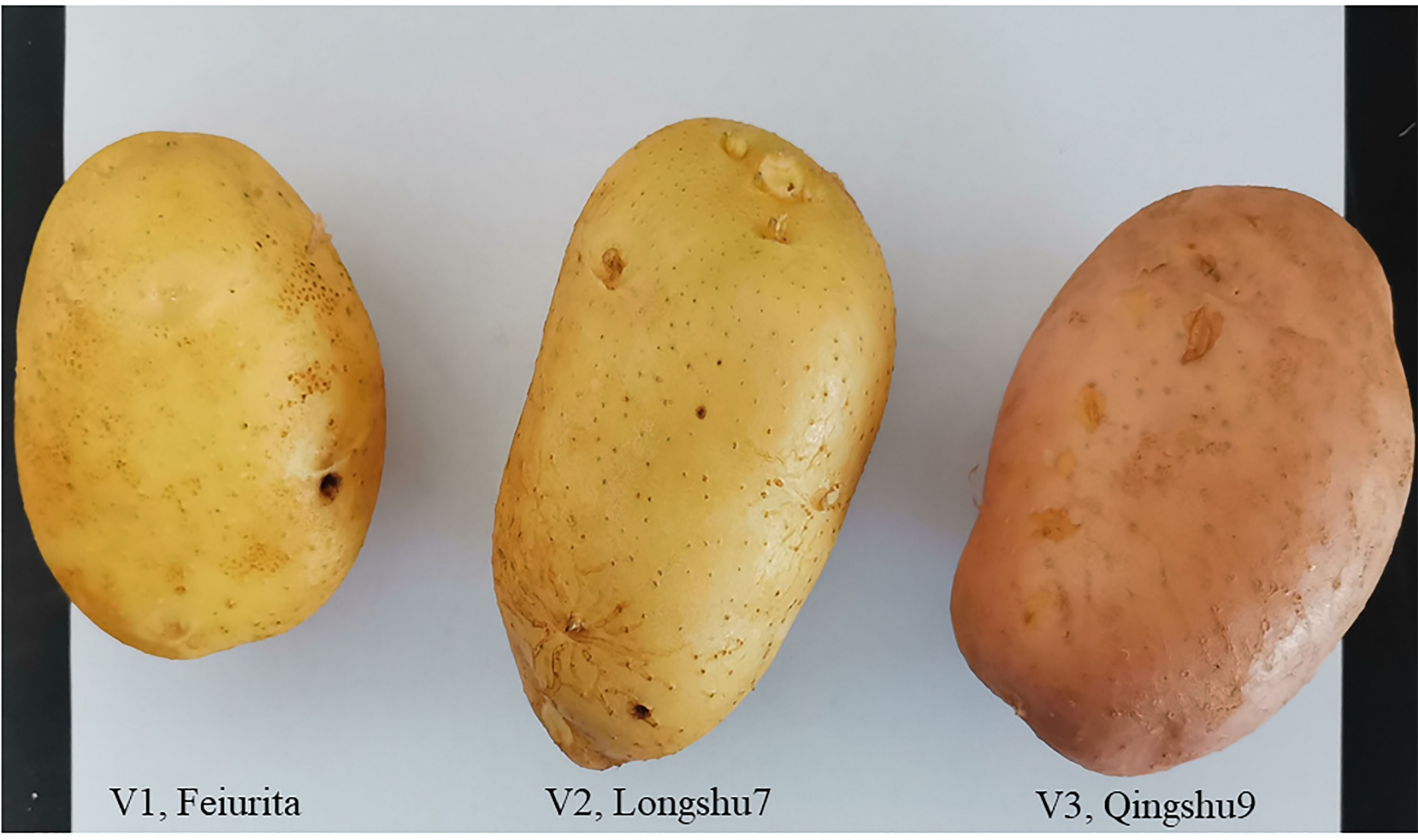
The three locally cultivated potato varieties.

The orthogonal experimental design encompassed nine treatments with three factors (irrigation amount × fertilizer rate × variety) and three levels. The three irrigation levels were W1 (100%ET_C_; ET_C_ is the crop evapotranspiration), W2 (80%ET_C_) and W3 (60%ET_C_). The three fertilizer (N-P_2_O_5_-K_2_O) levels were F1 (240‐120‐300 kg ha^−1^), F2 (180–90–225 kg ha^−1^) and F3 (120–60–150 kg ha^−1^). The proportion of N-P_2_O_5_-K_2_O was 1:0.5:1.25. The three locally cultivated potato varieties were V1 (Feiurita), V2 (Longshu 7) and V3 (Qingshu 9) (**Figure 1**). The nine treatments were T1 (W1F1V1), T2 (W1F2V2), T3 (W1F3V3), T4 (W2F1V2), T5 (W2F2V3), T6 (W2F3V1), T7 (W3F1V3), T8 (W3F2V1) and T9 (W3F3V2). Each treatment was repeated three times. Each plot was 3.6 m long and 3.3 m wide (3.6 m × 3.3 m = 11.88 m^2^). The experiment consisted of nine plots with a total area of 106.9 m^2^. A 1-meter-deep isolation ridge was set between every two plots to avoid interaction between different treatments.

Field trials were conducted during the potato growing season in 2022. The potatoes were planted on March 25 and harvested on July 25. The potato growth period was 122 days. The planting method was mechanical ridging, which was local traditional potato planting method. Potatoes were planted on ridges, and each ridge owned one row. The potato plants were sown at 20 cm intervals within each row, row spacing of 90 cm, and planting depth of 7–10 cm was adopted. Each plot was planted with three rows of potatoes, with a density of 45,455 plants ha^−1^. The ridge was covered with a white plastic film (1.2 m wide and 0.008 mm thick) to reduce evaporation and increase soil temperature. A weather station was installed in the greenhouse 2-meters away from the ground to record the temperature, humidity, atmospheric pressure, solar radiation and photosynthetic active radiation at 15-minute intervals. A thin-wall labyrinth drip irrigation tube with a diameter of 16 mm was adopted for irrigation. The spacing of drip irrigation emitters was 0.3 m. Ball valves and water meters were used to control the irrigation amounts of each plot.

The nitrogen fertilizer (Urea, N: 46%), phosphate fertilizer (Calcium superphosphate, P_2_O_5_: 12%) and potassium fertilizer (Potassium sulphate, K_2_O: 52%) were used as fertilizers, which were applied five times throughout the growing season. The proportion of fertilization was 1 (the germination and seedling stage, S1): 2 (the tuber formation stage, S2): 3 (the tuber enlargement stage, S3): 3 (the starch accumulation stage, S4): 1 (the tuber maturity stage, S5). The differential pressure tanks were used for fertilizer application in this experiment, and the capacity of each tank was 15 L. The solid fertilizers were fully dissolved in water before each fertilization. The pressure tank was set up in each plot and the same amount of fertilizer was applied. In the whole growth period of potatoes, the local field management methods were used to manage weeds and pests. After the potatoes were harvested, the drip irrigation pipes and plastic films were collected and recycled, and then Alfalfa was planted in all plots to balance the nutrient residues between the different treatments. The ET_C_ under different irrigation regimes was equal to the reference crop water requirement (ET_0_) multiplied by the potato crop coefficient (Kc), that is, ET_C_ = KcET_0_ (Kc was 0.5, 0.65, 1.15, 1.15 and 0.75 at S1, S2, S3, S4 and S5, respectively. The Penman-Monteith formula was used to calculate the reference crop evapotranspiration in the sandy area (Li et al., 2015).

### 2.3 Measurements and calculations

#### 2.3.1 Potato tuber yield

At harvest, the fresh tuber weight per plant, largest tuber weight and commodity tubers (individual tuber weight ≥ 75 g) were weighed on an area of 1.8m (width) × 3.3m (length) and 3 times per plot. The tuber yield of the plot and hectare was then calculated.

#### 2.3.2 Soil physical and chemical indicators

During potato harvest, three points in each plot were randomly selected to take soil samples, and were collected every 10 cm up to 60 cm. Soil samples were naturally dried and screened for use. PH was measured by a portable pH meter with thunder magnetic. The electrical conductivity (SEC) was measured by a lightning magnetic portable conductivity meter. The moisture content (SWC) was determined by drying weighing method. The content of nitrate nitrogen (SNNC) and available phosphorus (AP) was determined by ultraviolet spectrophotometer. Ammonium nitrogen content (SANC) was determined by indophenol blue colorimetry. The content of SAHC was determined by diffusion method. The content of AK was determined by flame spectrophotometry. Organic matter content (SOM) was determined by external heating method of concentrated sulfuric acid-potassium dichromate (Xing et al., 2020; Xing et al., 2022).

#### 2.3.3 Soil enzyme activity

In the S1, S2, S3, S4 and S5 periods, The collecting method of soil samples was the same as that of physical and chemical properties. The catalase activity (SCA) was determined by potassium permanganate titration. Alkaline phosphatase activity (SPA) was determined by colorimetric method. Urease activity (SUA) was determined by colorimetric method of sodium phenol-sodium hypochlorite. Sucrose activity (SSA) was determined by 3,5-dinitrosalicylic acid colorimetry (Xing et al., 2022).

### 2.4 Data analysis

Excel 2010 was used to sort out and analyze the test data. SPSS 23.0 software was used to conduct variance analysis on the effects of different fertilizer application amounts, irrigation amounts and varieties on various indexes under drip irrigation fertilization mode. Tukey’s HSD test at 95% confidence level (*P* < 0.05). Bivariate correlation analysis was used to evaluate the effects of fertilization, irrigation amount and variety on the relationship between each index. The graphics were created by Origin 9.0 software. Variable importance for projection (VIP) is a multivariate screening method, which calculates the contribution of independent variables to the prediction model and screens the independent variables according to their contribution degree. In this study, SIMCA 14.1 software was used to evaluate the soil factors affecting the yield of potato tuber by VIP.

## 3 Results

### 3.1 Soil water content and soil physicochemical indicators

**Table 1** shows that there were significant differences in soil fertility among the treatments at potato maturity stage(S5) (*P < *0.05). Except pH and moisture content (SWC), the contents of the other indexes in each treatment were 0~20 cm >20~40 cm >40~60 cm soil layer. The SWC, nitrate nitrogen content(SNNC) and organic matter content (SOM) of W1F1V1 (T1) were the highest, which were 2.41–35.05%, 5.37–88.97% and 8.38–38.33% higher than those of the other treatments, respectively. The amount of irrigation, fertilizer application and variety had significant effects on soil pH (*P < *0.01). The pH of W3 level was 2.41% and 4.25% higher than that of W1 and W2, respectively. With the increase of fertilizer application, soil pH decreased and the pH of F1 level was 1.39% and 4.36% lower than that of F2 and F3, respectively. The pH of V2 was 1.88% and 2.30% higher than that of V1 and V3, respectively.

**Table 1:** The pH, soil electrical conductivity (SEC), soil water content (SWC), soil nitrate-N content (SNNC), soil ammonium-N content (SANC), soil available potassium (AK), soil available phosphorus (AP), soil alkali-hydrolyzable nitrogen content (SAHC), soil organic matter (SOM) in different irrigation amount (W), fertilizer rates (F) and potato varieties (V) at the tuber maturity stage.

Treatments	Soil layer (cm)	pH	SEC (US cm^−1^)	SWC (%)	SNNC (mg kg^−1^)	SANC (mg kg^−1^)	AK (mg kg^−1^)	AP (mg kg^−1^)	SAHC (mg kg^−1^)	SOM (g kg^−1^)
T1	0–20	8.10 d	407.67 ab	9.27 a	130.54 a	3.02 c	131.01 ab	23.18 bc	44.74 ab	8.13 a
	20–40	8.14 de	314.83 abc	12.40 a	88.35 a	2.56 bc	121.83 ab	14.35 bc	31.01 abc	7.89 a
	40–60	8.34 c	173.22 abc	11.43 a	16.94 a	2.64 a	92.17 a	8.86 a	16.45 a	3.25 a
T2	0–20	8.53 b	162.63 c	7.73 b	120.84 a	2.92 c	136.33 a	24.73 bc	33.89 cd	8.41 a
	20–40	8.66 b	132.72 f	10.77 b	85.27 ab	2.55 bc	91.33 abc	16.68 abc	23.55 cde	6.35 abc
	40–60	8.74 b	99.38 c	11.23 ab	15.75 ab	2.82 a	64.17 c	7.00 b	12.05 ab	2.92 a
T3	0–20	8.29 c	359.67 b	9.08 a	105.66 ab	2.26 d	121.83 abc	21.19 c	31.53 cd	6.27 c
	20–40	8.48 c	244.60 cde	12.73 a	77.27 ab	2.07 c	109.23 abc	17.72 abc	19.08 de	4.65 c
	40–60	8.64 b	150.83 abc	10.51 bcd	15.15 ab	2.14 a	83.00 ab	5.59 c	11.26 b	3.01 a
T4	0–20	7.98 d	577.67 a	9.10 a	120.97 a	2.97 c	133.83 a	22.65 bc	39.11 bc	7.25 ab
	20–40	8.27 d	297.32 abcd	11.98 a	87.56 ab	2.52 bc	125.13 ab	18.59 ab	32.26 ab	6.27 abc
	40–60	8.33 c	212.48 a	10.88 abc	14.79 ab	2.46 a	95.00 a	6.55 bc	15.78 ab	3.87 a
T5	0–20	7.70 e	356.17 b	7.66 b	118.18 ab	3.02 c	136.83 a	20.40 c	30.71 cd	6.36 c
	20–40	8.09 e	169.40 ef	10.05 bc	80.79 ab	2.80 ab	100.33 abc	13.71 c	26.13 bcd	6.36 abc
	40–60	8.42 c	109.45 bc	11.66 a	14.58 ab	2.39 a	70.50 bc	7.75 ab	14.01 ab	3.44 a
T6	0–20	8.32 c	359.33 b	6.97 b	94.58 b	2.59 cd	111.67 bc	21.90 bc	28.03 d	7.22 ab
	20–40	8.67 b	200.58 def	9.46 cd	73.53 ab	2.68 b	67.67 c	14.73 bc	15.93 e	6.18 abc
	40–60	8.80 b	191.62 a	10.28 cd	13.14 b	2.69 a	63.17 c	7.08 b	11.23 b	3.32 a
T7	0–20	8.32 c	513.50 ab	6.91 b	128.65 a	3.85 a	135.00 a	30.52 a	37.80 bc	6.45 c
	20–40	8.48 c	351.17 ab	7.95 e	81.97 ab	3.24 a	95.33 abc	18.31 ab	35.03 a	6.42 abc
	40–60	8.64 b	164.40 abc	9.65 de	13.14 b	2.66 a	72.67 bc	7.80 ab	15.24 ab	3.99 a
T8	0–20	8.30 c	523.33 ab	6.97 b	125.90 a	3.58 ab	124.02 abc	27.21 ab	49.76 a	7.96 a
	20–40	8.44 c	394.50 a	8.79 de	83.53 ab	2.7a ab	142.17 a	20.39 a	33.80 ab	6.88 ab
	40–60	8.77 b	218.62 a	9.36 e	14.39 ab	2.05 a	71.67 bc	5.20 c	12.70 ab	2.94 a
T9	0–20	8.69 a	574.50 a	6.87 b	117.78 ab	3.10 bc	105.17 c	23.91 bc	32.11 cd	7.54 ab
	20–40	9.01 a	262.15 bcde	8.22 e	69.29 b	3.07 ab	89.37 bc	15.40 bc	22.34 de	4.99 bc
	40–60	9.10 a	188.77 ab	9.92 de	13.91 b	2.21 a	62.67 c	7.60 ab	13.09 ab	3.18 a
	Significance test									
	W	**	**	**	ns	**	ns	**	**	ns
	F	**	**	**	**	**	**	*	**	**
	V	**	ns	ns	ns	ns	ns	ns	ns	**

Different letters indicate significant difference at *P* < 0.05. ** indicates a remarkably significant difference (*P* < 0.01), * indicates a significant difference (*P* < 0.05), and ns indicates no significant difference (*P* > 0.05).

The amount of irrigation and fertilizer application had significant effects on soil electrical conductivity (SEC) (*P < *0.01). With the increase of irrigation amount, SEC showed a decreasing trend, and the SEC of W3 level was 55.99% and 28.98% higher than that of W1 and W2, respectively. The SEC of F1 level is 39.06% and 18.97% higher than that of F2 and F3, respectively. The amount of irrigation and fertilizer application had significant effects on SWC (*P <* 0.01). A “U” shaped trend was observed between fertilizer application and SWC, with the lowest SWC at F2 level. However, SWC showed an upward trend with the increase of irrigation, and SWC at W1 level was 8.08% and 27.48% higher than that at W2 and W3, respectively. The amount of fertilizer had a significant effect on SNNC and soil available potassium (AK) (*P <* 0.01). With the increase of fertilizer application rate, SNNC and AK showed an upward trend, and the SNNC and AK of F1 level were 3.29% and 6.89% higher than those of F2, and 35.46% and 23.13% higher than those of F3, respectively. The amount of irrigation and fertilizer application had significant effects on SANC and SAHC (*P < *0.01). With the increase of fertilizer application, the SANC and SAHC showed an upward trend, and the SANC and SAHC of F1 level were 4.39% and 13.03% higher than those of F2, and 13.63% and 44.86% higher than those of F3, respectively. The SANC and SAHC at W3 level were 15.14% and 12.66% higher than those at W1, and 9.70% and 18.14% higher than those at W2, respectively. The amount of irrigation had a significant effect on soil available phosphorus (AP) (*P <* 0.01), and the amount of fertilizer had a significant effect on AP (*P <* 0.05). With the increase of fertilizer application, AP showed an upward trend, and AP of F1 level was 5.41% and 11.61% higher than that of F2 and F3, respectively. With the increase of irrigation amount, AP decreased first and then increased, and the AP of W2 level was 4.26% and 14.70% lower than that of W1 and W3, respectively. The amount and variety of fertilizer had significant effect on SOM (*P < *0.01). The SOM of F1 level was 3.68% and 15.44% higher than that of F2 and F3, and the SOM of variety V1 was 5.89% and 14.53% higher than that of V2 and V3, respectively.

### 3.2 Soil enzyme activity

#### 3.2.1 Soil catalase activity

As shown in **Table 2**, there was a significant difference in soil catalase activity (SCA) among treatments (*P* < 0.05). The individual factors of irrigation amount and fertilization significantly affected SCA at the germination stage (S1), tuber enlargement stage (S3) and S5 (*P* < 0.01).

**Table 2:** The soil catalase activity (SCA (0.1 mol L^−1^ KMnO_4_ g^−1^ h^−1^)) in different irrigation amounts (W), fertilizer rates (F), and potato varieties (V) during S1 (the germination and seedling stage), S2 (the tuber formation stage), S3 (the tuber enlargement stage), S4 (the starch accumulation stage), and S5 (the tuber maturity stage).

Treatments	Soil layer (cm)	S1	S2	S3	S4	S5
T1	0–20	3.61 b	3.46 ab	3.26 a	3.08 ab	3.10 bcd
	20–40	3.67 c	3.81 a	3.41 bc	3.22 abcd	3.26 abc
	40–60	5.12 ab	3.95 a	3.64 a	3.36 a	3.11 a
T2	0–20	4.26 ab	3.43 abcd	3.28 b	3.23 a	3.19 abc
	20–40	4.56 a	3.68 ab	3.76 a	3.29 abc	3.52 a
	40–60	5.10 ab	3.79 a	3.31 c	3.21 a	3.19 a
T3	0–20	4.14 ab	3.65 a	3.18 ab	2.94 ab	3.03 bcd
	20–40	3.87 bc	3.68 ab	3.57 abc	2.96 d	3.22 abcd
	40–60	4.45 cd	3.75 a	3.39 bc	3.04 ab	3.20 a
T4	0–20	3.98 ab	3.44 abc	3.19 ab	3.08 ab	2.85 de
	20–40	4.34 ab	3.85 a	3.53 abc	3.12 cd	3.14 bcd
	40–60	5.18 ab	4.13 a	3.69 a	3.07 ab	3.16 a
T5	0–20	4.77 a	3.35 abcd	3.15 ab	3.36 a	3.41 a
	20–40	4.56 a	3.57 abc	3.67 ab	3.49 a	3.38 ab
	40–60	5.29 a	4.15 a	3.69 a	3.41 a	3.30 a
T6	0–20	3.77 b	3.02 d	2.99 bc	3.07 ab	3.23 ab
	20–40	4.23 abc	3.27 c	3.56 abc	3.45 ab	3.54 a
	40–60	4.69 abc	3.80 a	3.56 ab	3.09 ab	3.32 a
T7	0–20	3.86 b	3.03 cd	3.00 bc	2.52 b	2.82 de
	20–40	4.50 ab	3.47 abc	3.38 c	3.17 bcd	2.94 cd
	40–60	4.61 bc	3.68 a	3.40 bc	2.96 b	3.11 a
T8	0–20	4.34 ab	3.31 abcd	3.02 bc	3.05 ab	2.91 cde
	20–40	4.64 a	3.78 a	3.54 abc	3.08 cd	2.97 cd
	40–60	4.39 cd	3.88 a	3.30 c	3.14 ab	3.21 a
T9	0–20	3.65 b	3.06 bcd	2.86 c	3.02 ab	2.66 e
	20–40	3.66 c	3.30 bc	3.31 c	3.32 abc	2.87 d
	40–60	3.99 d	3.77 a	3.40 bc	3.17 ab	3.09 a
	Significance test					
W		**	ns	**	ns	**
F		**	ns	**	ns	*
V		ns	ns	ns	ns	ns

Different letters indicate significant difference at *P* < 0.05. ** indicates a remarkably significant difference (*P* < 0.01), * indicates a significant difference (*P* < 0.05), and ns indicates no significant difference (*P* > 0.05).

Potato variety had no significant effects on SCA during various growth periods (*P* < 0.05). SCA among treatments showed 40–60 cm soil layer > 20–40 cm soil layer > 0–20 cm soil layer. SCA in various treatments decreased gradually from S1 to the S5 and reached the highest value at S1. Irrigation amount and fertilization had different effects on SCA during various growth periods. With the increase of fertilization, at S1, S3 and S5, SCA increased first and then decreased, and F2 treatment increased by 7.72%, 0.78% and 5.78% compared with F1 at three growth stages, respectively.

At S1, S3 and S5, SCA increased first and then decreased, and F2 treatment increased by 7.72%, 0.78% and 5.78% compared with F1 at three growth stages, respectively. When the fertilization and variety were the same, SCA increased first and then decreased with the increase in irrigation amount. Compared with W1 and W3, the average SCA under W2 was increased by 1.84% and 7.04%, respectively. The difference in SCA among the three varieties was small during various growth periods.

#### 3.2.2 Soil phosphatase activity

The irrigation amount had a significant effect on soil phosphatase activity (SPA) at S1, S2 and S3 (*P* < 0.01) (**Table 3**). The individual factors of irrigation amount and fertilization significantly affected SPA at the starch accumulation stage (S4) (*P* < 0.05).

**Table 3:** The SPA (mg g^−1^ 24 h^−1^) in different irrigation amounts (W), fertilizer rates (F), and potato varieties (V) during S1, S2, S3, S4, and S5.

Treatments	Soil layer (cm)	S1	S2	S3	S4	S5
T1	0–20	0.38 a	0.39 ab	0.28 a	0.28 abc	0.26 abc
	20–40	0.21 a	0.26 ab	0.23 ab	0.16 b	0.21 a
	40–60	0.12 a	0.14 a	0.11 ab	0.09 ab	0.09 a
T2	0–20	0.35 ab	0.37 abc	0.27 ab	0.29 ab	0.23 bcd
	20–40	0.21 a	0.27 ab	0.24 a	0.17 b	0.20 a
	40–60	0.12 a	0.15 a	0.12 a	0.10 a	0.09 a
T3	0–20	0.34 abc	0.34 bcd	0.26 ab	0.25 bc	0.25 abcd
	20–40	0.19 a	0.26 ab	0.24 a	0.17 b	0.19 a
	40–60	0.13 a	0.13 a	0.10 bc	0.08 ab	0.09 a
T4	0–20	0.33 abc	0.34 bcd	0.28 a	0.26 bc	0.27 ab
	20–40	0.17 a	0.26 ab	0.22 abc	0.16 b	0.21 a
	40–60	0.11 a	0.16 a	0.12 a	0.09 ab	0.10 a
T5	0–20	0.38 a	0.41 a	0.26 ab	0.32 a	0.29 a
	20–40	0.20 a	0.27 a	0.25 a	0.22 a	0.22 a
	40–60	0.11 a	0.15 a	0.13 a	0.08 ab	0.10 a
T6	0–20	0.34 abc	0.39 ab	0.24 ab	0.29 abc	0.21 cd
	20–40	0.21 a	0.27 ab	0.24 a	0.16 b	0.18 a
	40–60	0.11 a	0.15 a	0.12 a	0.09 ab	0.10 a
T7	0–20	0.29 c	0.34 bcd	0.20 b	0.24 c	0.21 d
	20–40	0.18 a	0.23 b	0.20 bc	0.15 b	0.17 a
	40–60	0.10 a	0.14 a	0.07 d	0.09 ab	0.09 a
T8	0–20	0.29 c	0.29 d	0.24 ab	0.24 bc	0.26 abc
	20–40	0.17 a	0.23 b	0.19 c	0.17 b	0.18 a
	40–60	0.09 a	0.15 a	0.09 cd	0.08 ab	0.09 a
T9	0–20	0.32 bc	0.32 cd	0.24 ab	0.26 bc	0.23 bcd
	20–40	0.17 a	0.24 ab	0.19 bc	0.20 ab	0.19 a
	40–60	0.09 a	0.13 a	0.07 d	0.07 b	0.09 a
	Significance test					
W		**	**	**	*	ns
F		ns	ns	ns	*	ns
		ns	ns	ns	ns	ns

Different letters indicate significant difference at *P* < 0.05. ** indicates a remarkably significant difference (*P* < 0.01), * indicates a significant difference (*P* < 0.05), and ns indicates no significant difference (*P* > 0.05).

There was a significant difference in SPA among treatments (*P* < 0.05). The SPA increased first and then decreased during the growth period, and reached the highest value in S2. SPA among treatments showed 0–20 cm > 20–40 cm > 40–60 cm soil layer. Compared with the 20–40 cm and 40–60 cm soil layer, the average SPA under the 0–20 cm was increased by 41.39% and 173.05%, respectively. With the increase of fertilization, SPA increased first and then decreased. Compared with F1 and F3, the average SPA under F2 was increased by 3.94% and 4.51%, respectively. At S4, when the fertilization and variety were the same, SPA increased first and then decreased with the increase of irrigation amount. Averaging across all irrigation amounts and fertilizer rates, the SPA in V3 was 3.59% higher than that in V1, but the difference between V3 and V2 was small.

#### 3.2.3 Soil urease activity

As shown in **Table 4**, the irrigation amount had a significant effect on soil urease activity (SUA) during various growth periods (*P* < 0.05). The individual factors of fertilization significantly affected SUA at S4 (*P*< 0.05). However, the potato variety had no significant effects on SUA during various growth periods (*P* > 0.05). SUA among treatments had a significant difference (*P* < 0.05), and showed 0–20 cm > 20–40 cm > 40–60 cm soil layer. The SUA increased first and then decreased during the growth period, and reached the highest value in S2 among treatments. Compared with F1 and F3, the average SUA under F2 was increased by 3.51% and 3.76%, respectively. Averaging across all fertilization and variety, the SUA in W2 was 8.26% and 9.62% higher than that in W1 and W3, respectively. However, the difference in SUA among the three varieties was small during various growth periods.

**Table 4:** The SUA (mg g^−1^ 24 h^−1^) in different irrigation amounts (W), fertilizer rates (F), and potato varieties (V) during S1, S2, S3, S4, and S5.

Treatments	Soil layer (cm)	S1	S2	S3	S4	S5
T1	0–20	0.42 abc	0.67 abc	0.57 ab	0.46 b	0.49 ab
	20–40	0.30 ab	0.41 ab	0.33 b	0.36 a	0.29 a
	40–60	0.26 ab	0.12 a	0.09 a	0.11 ab	0.11 ab
T2	0–20	0.43 abc	0.68 ab	0.60 ab	0.49 ab	0.51 ab
	20–40	0.33 ab	0.41 ab	0.36 ab	0.38 a	0.28 a
	40–60	0.25 ab	0.14 a	0.10 a	0.12 a	0.09 ab
T3	0–20	0.42 abc	0.64 bc	0.59 ab	0.47 b	0.51 ab
	20–40	0.30 b	0.36 b	0.34 b	0.36 a	0.23 a
	40–60	0.23 b	0.11 a	0.08 a	0.10 bc	0.11 ab
T4	0–20	0.43 ab	0.74 a	0.60 ab	0.50 ab	0.55 a
	20–40	0.35 ab	0.39 ab	0.36 ab	0.36 a	0.31 a
	40–60	0.28 ab	0.15 a	0.08 a	0.12 a	0.11 ab
T5	0–20	0.43 ab	0.74 a	0.61 a	0.54 a	0.52 ab
	20–40	0.34 ab	0.43 a	0.41 a	0.43 a	0.32 a
	40–60	0.30 a	0.16 a	0.09 a	0.12 ab	0.11 ab
T6	0–20	0.44 a	0.70 ab	0.60 ab	0.51 ab	0.52 ab
	20–40	0.36 a	0.44 a	0.41 a	0.42 a	0.29 a
	40–60	0.27 ab	0.14 a	0.08 a	0.13 a	0.11 a
T7	0–20	0.38 bc	0.66 bc	0.51 b	0.47 b	0.46 b
	20–40	0.32 ab	0.42 ab	0.33 b	0.38 a	0.27 a
	40–60	0.26 ab	0.15 a	0.09 a	0.11 abc	0.10 ab
T8	0–20	0.38 bc	0.67 abc	0.53 ab	0.54 a	0.49 ab
	20–40	0.31 ab	0.41 ab	0.40 a	0.39 a	0.24 a
	40–60	0.26 ab	0.14 a	0.07 a	0.10 bc	0.10 ab
T9	0–20	0.38 c	0.61 c	0.53 ab	0.49 ab	0.45 b
	20–40	0.30 b	0.44 a	0.42 a	0.40 a	0.25 a
	40–60	0.26 ab	0.12 a	0.09 a	0.09 c	0.09 b
	Significance test					
W		**	**	*	**	**
F		ns	ns	ns	*	ns
V		ns	ns	ns	ns	ns

Different letters indicate significant difference at *P* < 0.05. ** indicates a remarkably significant difference (*P* < 0.01), * indicates a significant difference (*P* < 0.05), and ns indicates no significant difference (*P* > 0.05).

#### 3.2.4 Soil sucrase activity

There was a significant difference in soil sucrase activity (SSA) among treatments (*P* < 0.05) (**Table 5**). The irrigation amount had a significant effect on SSA at S1, S2, S3 and S5 (*P* < 0.01). The individual factors of fertilization significantly affected SSA at S1 and S5 (*P* < 0.01). Compared with the 20–40 cm and 40–60 cm soil layer, the average SSA under the 0–20 cm soil layer was increased by 21.11% and 77.32%, respectively. The SSA reached the highest value in S2 among treatments. Compared with V2 and V3, the average SSA under V1 was increased by 2.33% and 1.10%, respectively. When the fertilization and variety were the same, SSA increased first and then decreased with the increase in irrigation amount. Compared with W1 and W3, the average SSA under W2 increased by 5.34% and 13.36%, respectively. With the increase of fertilization, SSA increased first and then decreased, and reached the highest value in F2.

**Table 5:** The SSA in different irrigation amounts (W), fertilizer rates (F), and potato varieties (V) during S1, S2, S3, S4, and S5.

Treatments	Soil layer (cm)	S1	S2	S3	S4	S5
T1	0–20	17.96 ab	24.96 bc	20.54 b	21.22 abc	16.10 abc
	20–40	14.87 ab	17.39 d	17.79 a	16.09 ab	13.58 bc
	40–60	11.60 a	13.63 ab	9.74 a	12.38 a	10.77 ab
T2	0–20	19.76 a	28.25 ab	17.21 cd	22.45 abc	18.48 a
	20–40	18.47 a	23.70 ab	16.13 a	14.81 bc	13.05 c
	40–60	10.65 a	13.16 ab	9.94 a	12.41 a	9.90 b
T3	0–20	17.36 b	25.19 bc	19.36 bc	20.73 abc	12.96 d
	20–40	17.01 ab	19.98 bcd	16.58 a	13.78 c	14.06 abc
	40–60	12.27 a	12.89 ab	10.42 a	12.76 a	10.01 ab
T4	0–20	17.22 b	23.55 c	23.34 a	24.35 a	15.44 bc
	20–40	15.44 ab	19.78 bcd	16.57 a	16.10 ab	15.23 a
	40–60	12.59 ab	12.25 ab	9.56 a	12.52 a	10.92 ab
T5	0–20	16.02 bc	30.18 a	23.57 a	24.85 a	18.20 a
	20–40	14.49 ab	22.76 abc	17.82 a	16.85 a	14.95 ab
	40–60	11.36 a	13.82 a	9.55 a	13.22 a	10.59 ab
T6	0–20	20.10 a	29.93 a	17.86 bcd	24.02 ab	17.81 ab
	20–40	17.15 ab	24.41 a	17.99 a	15.67 abc	13.62 bc
	40–60	11.67 a	13.70 ab	10.12 a	13.20 a	10.59 ab
T7	0–20	16.44 bc	23.48 c	16.41 d	18.98 bc	16.11 abc
	20–40	15.79 ab	20.41 abcd	16.96 a	14.36 bc	13.25 c
	40–60	11.26 a	10.49 b	10.37 a	10.05 a	10.39 ab
T8	0–20	16.26 bc	24.80 bc	19.04 bcd	18.70 c	13.99 cd
	20–40	15.04 ab	20.77 abcd	17.79 a	14.27 bc	13.78 bc
	40–60	11.84 a	10.86 ab	10.35 a	11.02 a	11.37 a
T9	0–20	14.21 c	25.05 bc	16.61 cd	18.27 c	14.30 cd
	20–40	13.59 b	18.77 cd	15.44 a	15.25 abc	12.84 c
	40–60	11.89 a	11.17 ab	8.99 a	10.33 a	9.89 b
	Significance test					
W		*	**	**	**	**
F		ns	**	ns	ns	**
V		ns	ns	ns	ns	ns

Different letters indicate significant difference at *P* < 0.05. ** indicates a remarkably significant difference (*P* < 0.01), * indicates a significant difference (*P* < 0.05), and ns indicates no significant difference (*P* > 0.05).

### 3.3 Potato tuber yield

**Figure 2:**
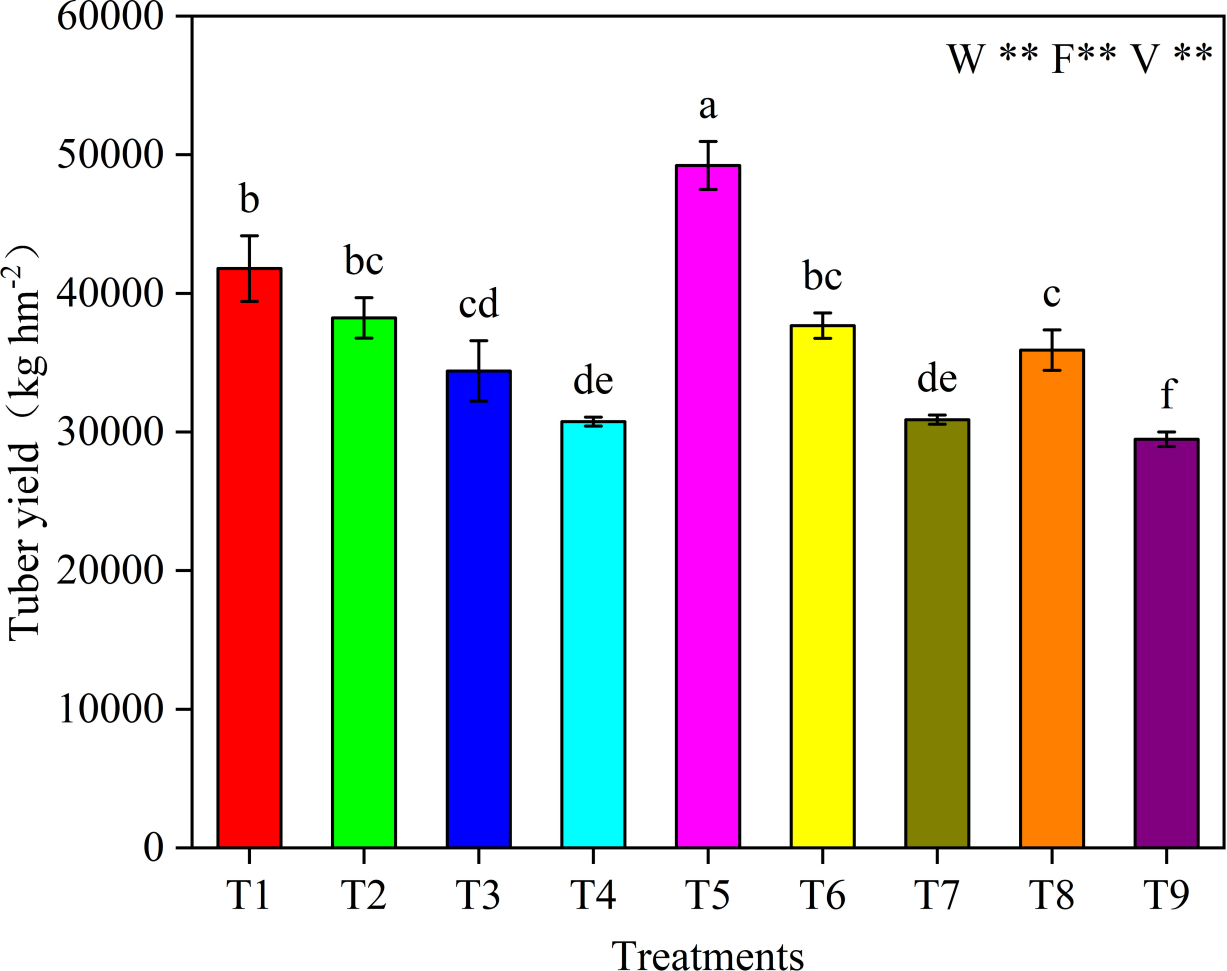
Potato tuber yield in different irrigation amounts (W), fertilizer rates (F) and potato varieties (V) at the tuber maturity stage. Different letters above the bars indicate significant difference at *P* < 0.05. ** indicates a remarkably significant difference (*P* < 0.01).

Fertilization rate, irrigation rate and variety had significant effects on potato yield (*P* < 0.01) (**Figure 2**). There were significant differences in yield among treatments (*P* < 0.05). The highest yield of T5 was 49,222.33 kg hm^−2^.

The yield of variety V3 was 16.31% higher than that of variety V2, but the difference between variety V1 and V3 was small. With the increase of irrigation amount, the yield increased first and then decreased. The yield under W2 level was 2.81% and 22.20% higher than that of W1 and W3, respectively. Appropriate fertilization can improve potato yield, while excessive fertilization will result in yield reduction. The yield under F2 level is 19.28% and 21.48% higher than that of F1 and F3, respectively.

### 3.4 Correlation analysis

**Figure 3:**
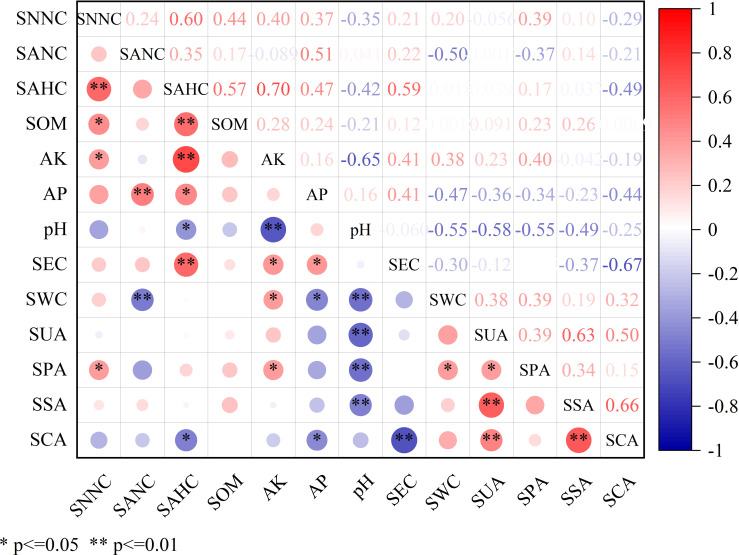
The correlation analysis between soil fertility and soil enzyme activity at tuber maturity stage. * means significant correlation at 0.05 level, ** means significant correlation at 0.01 level. SNNC, soil nitrate-N content; SANC, soil ammonium-N content; SAHC, soil alkali-hydrolyzable nitrogen content; SOM, soil organic matter; AK, soil available potassium; AP, soil available phosphorus; pH, soil pH value; SEC, soil electrical conductivity; SWC, soil water content; SUA, soil urease activity; SPA, soil phosphatase activity; SSA, soil sucrase activity; SCA, soil catalase activity.

The correlation analysis between soil fertility and soil enzyme activity at the tuber maturity stage was shown in **Figure 3**. The soil factor SNNC had a significant influence on SAHC, SOM, AK and SPA, and the correlation coefficients were 0.60, 0.44, 0.40 and 0.39, respectively. The soil factor SANC had a significant influence on AP and SWC, and the correlation coefficients were 0.51 and −0.50, respectively. The soil factor SAHC had a significant influence on SOM, AK, AP, pH, SEC and SCA, and the correlation coefficients were 0.57, 0.70, 0.47, −0.42, 0.59 and −0.49, respectively. The soil factor AK had a significant influence on pH, SEC, SWC and SPA, and the correlation coefficients were −0.66, 0.41, 0.38 and 0.40, respectively. The soil factor AP had a significant influence on SEC, SWC and SCA, and the correlation coefficients were 0.41, −0.47 and −0.44, respectively. The soil factor pH had a significant influence on SWC, SUA, SPA and SSA, and the correlation coefficients were −0.55, −0.58, −0.55 and −0.50, respectively. There was a significant correlation between SEC and SCA, SWC and SPA, SSA and SCA, and the correlation coefficients were −0.68, 0.66 and 0.39, respectively. The soil factor SUA had a significant influence on SPA, SSA and SCA, and the correlation coefficients were 0.39, 0.63 and 0.50, respectively.

### 3.5 Variable importance for projection of soil factors affecting the potato tuber yield

As shown in **Table 6**, the soil factors had a significant influence on potato tuber yield, and the correlation coefficients of pH, SEC, SPA, SSA and SCA were −0.56, −0.44, 0.40, 0.55 and 0.60, respectively. In this study, the variable importance for projection (VIP) value greater than 1.0 was used as the screening criterion to select the main soil factors that affecting potato tuber yield characteristics. SCA (VIP, 1.625), pH (VIP, 1.513), SSA (VIP, 1,510), SEC (VIP, 1.187), SPA (VIP, 1.100), SUA (VIP, 1.010) great influences on potato tuber yield.

**Table 6:** Variable importance for projection of soil factors affecting the potato tuber yield.

Indicators	Correlation coefficient	Variable importance in projection	Variable importance in projection order
SNNC	0.06	0.153	12
SANC	−0.15	0.398	10
SAHC	−0.02	0.057	13
SOM	0.26	0.701	9
AK	0.11	0.298	11
AP	−0.33	0.905	7
pH	−0.56**	1.513	2
SEC	−0.44*	1.187	4
SWC	0.28	0.752	8
SUA	0.37	1.010	6
SPA	0.40*	1.100	5
SSA	0.55**	1.510	3
SCA	0.60**	1.625	1

*means significant correlation at 0.05 level, **means significant correlation at 0.01 level. SNNC, soil nitrate-N content; SANC, soil ammonium-N content; SAHC, soil alkali-hydrolyzable nitrogen content; SOM, soil organic matter; AK, soil available potassium; AP, soil available phosphorus; pH, soil pH value; SEC, soil electrical conductivity; SWC, soil water content; SUA, soil urease activity; SPA, soil phosphatase activity; SSA, soil sucrase activity; SCA, soil catalase activity.

## 4 Discussion

The rational combination of irrigation and fertilizer can effectively increase potato yield (Li et al., 2018). Appropriate application of fertilizer can increase soil nutrients in root zone and increase crop yield (Xu et al., 2022). With the increase of fertilizer application, potato yield showed a continuously increasing trend(Gong et al., 2022; Zhang et al., 2022). This difference is mainly caused by the difference of soil texture and soil basic nutrients in different regions. In this study, with the increase of fertilizer application, potato yield and fertilizer application showed a unimodal trend, and the yield under F2 level was 19.28% and 21.48% higher than that of F1 and F3, respectively. The reasons for this result are as follows. First, fertilizer application is an important means to increase crop yield, but fertilizer application amount is not always linearly correlated with yield. Excessive fertilizer input will seriously damage soil micro-ecosystem in root zone, affect the normal growth and development of crop roots, limit the absorption and utilization of soil water and fertilizer, and result in production reduction. Second, fertilization can promote crop growth and accumulation of dry matter mass, but excessive fertilizer input will intensify the nutrient competition between plant vegetative organs and reproductive organs, which is not conducive to the formation of yield. Irrigation amount directly or indirectly affects crop growth and yield formation. In this study, the yield at W2 level was 2.81% and 22.20% higher than that at W1 and W3, respectively, indicating that deficit irrigation or excessive irrigation would affect crop growth and reduce crop yield. The reason for this result may be that when crops are under drought stress, the chloroplast and stomatal system of plant leaves will be damaged, the intensity of photosynthesis will be reduced, and the normal growth and development of crops cannot be ensured, resulting in serious yield reduction (Martínez-Goñi et al., 2022; Bouremani et al., 2023). However, when the irrigation amount is too high, many nutrients will be leached out of the rhizosphere, and the ventilation condition around the rhizosphere will be affected, and which further influence yield (Wang et al., 2021a). A reasonable amount of irrigation can make a large amount of water infiltrate around the roots, ensure the continuous supply of water and fertilizer around the roots of crops, reduce the leaching of nutrients, and improve the yield. Variety is another major factor affecting crop yield. Cultivar has significant influence on nitrogen, phosphorus and potassium absorption and yield of potato in newly cultivated land (Piekutowska et al., 2021). In this study, varieties had a very significant effect on the formation of potato yield (*P <* 0.01), and the yield of varieties Qingshu 9 and Feiurita was significantly higher than that of Longshu 7. The reason of this phenomenon may be related to variety characteristics, climate environment and farming management. The correlation analysis and partial least squares analysis showed that soil nutrient factors and enzyme activities were closely related to tuber yield, and root zone SCA, pH, SSA, SEC, SPA and SUA were the core factors affecting potato yield. Therefore, it is suggested to reduce fertilizer application, regulate soil pH and SEC in root zone, and activate enzyme activity in root zone in the later potato planting practice in the arid region of northern Shaanxi to achieve yield increase.

Different field management methods will produce different responses to root zone nutrients (Corwin, 2021). It was found that there were significant differences in soil fertility indexes among different treatments. SEC, SNNC, SANC, SAHC, AK, AP and SOM in the root zone decreased with the increase of soil depth, and the contents of various soil nutrient indexes were concentrated in the upper soil. The same study showed that water was the most important factor driving the dynamic changes of soil nutrients in the root layer (Zhang et al., 2018). In this study, SNNC, SANC and SAHC at the W1 level were significantly lower than those at the W3 level, indicating that adequate irrigation could alleviate the adverse effects of drought stress on crops and facilitate the uptake and utilization of soil nutrients by roots. Similar results showed that fertilizer was the decisive factor affecting soil fertility. Fertilization can improve soil nutrients in the root zone, promote crop growth and increase yield, but excessive fertilization will cause a large amount of nutrient loss and eventually cause serious environmental harm (Grzebisz et al., 2022). In this study, SEC, SNNC, SANC, AK, AP, SAHC and SOM tended to increase significantly with the increase of fertilizer application, which was mainly because the application of chemical fertilizer significantly increased the content of soil available nutrients, but at the same time, it also increased the risk of nutrient leaching loss. The influence of fertilizer application rate and irrigation rate on SWC in root zone was extremely significant. The influence of fertilizer application rate on SWC was the highest at F1 level and the lowest at F2 level, indicating that fertilizer application rate would directly affect the water and nutrient absorption capacity of crop roots. Crop root development would be seriously stressed under excessive fertilizer application rate, which reduced the absorption and utilization of water and fertilizer in the root zone. In addition, in this study, varieties had significant effects on soil pH and SOM in the root zone.

Soil enzymes exist widely in soil and are important organic components of soil (Wang et al., 2023). Soil enzyme activity was more active in the soil surface (Li et al., 2023). In this study, SPA, SUA and SSA of the root zone at different growth periods showed a trend of decrease with the increase of soil depth. However, SCA showed high activity in the 20–40 cm soil layer. This may be related to the species and quantity of soil microorganisms in the root zone, soil hydrothermal conditions and root growth secretions (Sun et al., 2022). In addition, the root zone SCA, SPA, SUA and SSA showed higher activity at S1 or S2. Catalase can promote the decomposition of hydrogen peroxide in soil and eliminate the toxic effect of hydrogen peroxide on crops. Alkaline phosphatase can promote the hydrolysis of phosphate ester or phosphoric anhydride, so that soil organic phosphate mineralization, the level of its activity reflects the nutrient status of soil phosphorus (Tian et al., 2016). Soil urease and sucrase are the key enzymes to promote soil nitrogen conversion and carbon cycle (Li et al., 2021). The results of this study showed that, under a certain threshold value, appropriate fertilization could significantly improve soil enzyme activity in the root zone. In this study, SCA, SPA, SUA and SSA at F2 level were significantly higher than those at F1 and F3. The reasons for this result may be as follows. First, appropriate fertilizer input will ensure the normal growth and development of crops, to provide rich carbon and nitrogen sources for the root layer soil, which is the main driving force for the improvement of soil enzyme activities in the root zone. Second, appropriate fertilizer input will optimize the crop root system, increase the amount of rhizosphere secretions, activate rhizosphere microbial activity, and indirectly improve soil enzyme activity in the root zone, thus increasing yield. Appropriately increasing irrigation is another way to improve soil enzyme activity in root zone. In this study, SCA, SUA and SSA at W2 level were 1.84% and 7.04%, 8.26% and 9.62%, 5.34% and 13.36% higher than W1 and W3, respectively. The reason for this result is that proper irrigation can ensure good nutrient supply and ventilation conditions around the roots, provide a favorable living environment for rhizosphere microorganisms, and thus increase soil enzyme activities in the root zone. In addition, the soil enzyme activity in the root zone of variety V3 was higher than that of varieties V1 and V2, but there was no significant difference between the three varieties. From the perspective of soil enzyme activity in the root zone, SCA, SPA, SUA and SSA in the root zone treated with T5 were superior to other treatments.

Correlation analysis showed that soil enzymes not only had specific properties, but also had some commonality. Rhizosphere urease and phosphatase not only show their own characteristics but also the same commonness in promoting the transformation of soil organic nutrients, material circulation and energy flow in soil, which jointly affect the improvement of soil fertility and increase millet yield (Trujillo-Narcía et al., 2019; Cui et al., 2023). In this study, SNNC was positively correlated with SOM, AK and AP, SPA was positively correlated with SNNC and AK, SPA was positively correlated with SUA, and SUA was positively correlated with SCA and SSA, all of which reached a significant level or extremely significant level, indicating that the higher the activity of these enzymes, the higher the soil nutrient content. These enzymes in common with each other can reflect soil fertility to some extent. In addition, in this study, the correlation between SUA in the root zone and SPA, SSA and SCA reached a significant level, a very significant level and a very significant level, respectively, and there was a close relationship between different soil enzyme activities in the root zone.

## 5 Conclusion

The yield, soil fertility and enzyme activity in root zone of different potato varieties were significantly affected by different water and fertilizer supplies. Tuber yield of T5 treatment was significantly higher than that of other treatments. SEC, SNNC, SANC, AK, AP, SAHC and SOM in potato root zone increased continuously with the increase of fertilizer application rate, while SWC decreased first and then increased. Soil pH, SEC, SANC and SAHC decreased with the increase of irrigation amount, while SWC content was positively correlated with irrigation amount. Variety had significant effects on pH and SOM in root zone. An appropriate amount of irrigation and fertilizer application could effectively improve the root zone SCA, SPA, SSA and SUA during the whole growth period, and the variety had no significant effect on the root zone soil enzyme activity, while T5 treatment had a higher root zone soil enzyme activity. Soil nutrients and enzyme activities were closely related, and there was significant correlation. Root zone SCA, pH, SSA, SEC, SPA and SUA are the core factors affecting potato yield. In conclusion, treatment of T5 (80%ET_C_, 180-90-225 kg hm^−2^ N-P_2_O_5_-K_2_O, Qingshu 9) under drip irrigation fertilization mode can not only improve soil micro-ecosystem, soil enzyme activity and soil nutrient cycling, but also achieve the high yield goal of green environmental protection and sustainable development. The highly efficient water and fertilizer management planting model can be recommended as the best variety of potato in arid regions of Northwest China.

## Data availability statement

The raw data supporting the conclusions of this article will be made available by the authors, without undue reservation.

## Author contributions

FZ and YX conceptualized this study, MC and JF designed the study, FZ and YX performed data checks, XZ and YL wrote the original draft preparation, FZ and YX edited and revised the original manuscript. All authors have read and agreed to the published version of the manuscript.
